# Absolute quantification of nicotinamide mononucleotide in biological samples by double isotope-mediated liquid chromatography-tandem mass spectrometry (dimeLC-MS/MS)

**DOI:** 10.1038/s41514-023-00133-1

**Published:** 2024-01-02

**Authors:** Junya Unno, Kathryn F. Mills, Tairo Ogura, Masayuki Nishimura, Shin-ichiro Imai

**Affiliations:** 1 Technology Research Laboratory, Shimadzu Corporation, Kyoto, Japan; 2 Department of Developmental Biology, Washington University School of Medicine, St. Louis, MO USA; 3 Innovation Center, Shimadzu Scientific Instruments, Inc., Columbia, MD USA; 4 New Strategy Department, Shimadzu Scientific Instruments, Inc., Columbia, MD USA

**Keywords:** Metabolomics, Biomarkers

## Abstract

Nicotinamide adenine dinucleotide (NAD^+^) is an essential metabolite for fundamental biological phenomena, including aging. Nicotinamide mononucleotide (NMN) is a key NAD^+^ intermediate that has been extensively tested as an effective NAD^+^-boosting compound in mice and humans. However, the accurate measurement of NMN in biological samples has long been a challenge in the field. Here, we have established an accurate, quantitative methodology for measuring NMN by using liquid chromatography-triple quadrupole mass spectrometry (LC-MS/MS) with double isotopic NMN standards. In this new methodology, the matrix effects of biological samples were properly adjusted, and the fate of NMN could be traced during sample processing. We have demonstrated that this methodology can accurately quantitate NMN levels in mouse plasma and confirmed quick, direct NMN uptake into blood circulation and cells. This double isotope-mediated LC-MS/MS (dimeLC-MS/MS) can easily be expanded to other NAD^+^-related metabolites as a reliable standard methodology for NAD^+^ biology.

## Introduction

NAD^+^ is a classic coenzyme for many fundamental redox reactions and also a substrate for NAD^+^-consuming enzymes including poly-ADP-ribose polymerases (PARPs), sirtuins, CD38/157, and SARM1, thereby playing a critical role in myriad of important biological processes including metabolism, DNA damage response, inflammation, cancer, neurodegeneration, and aging^1–4^. It has been shown that systemic reduction in NAD^+^ levels is an important cause of age-associated tissue dysfunctions and diseases^4–7^. Thus, supplementation methods of NAD^+^ intermediates, such as nicotinamide mononucleotide (NMN) and nicotinamide riboside (NR), have gained significant attention as promising anti-aging interventions in both the scientific community and general public^4,7–11^.

NAD^+^ is synthesized from three major precursors: tryptophan, nicotinic acid (NA), and nicotinamide (NAM), and also from two intermediates, NMN and NR^1–4^. The major NAD^+^ biosynthetic pathway in mammals starts from NAM, catalyzed by nicotinamide phosphoribosyltransferase (NAMPT)^12,13^. NAMPT converts NAM and 5’-phosphoribosyl pyrophosphate (5’-PRPP) into NMN, and NMN is then converted to NAD^+^ by NMN adenylyltransferases 1-3 (NMNAT1-3). NR needs to be phosphorylated to NMN by NR kinases 1 and 2 (NRK1 and 2) to enter the major NAD^+^ biosynthetic pathway. Whereas biochemical features of key NAD^+^ biosynthetic enzymes have been well characterized, the accurate measurement of NAD^+^-related metabolites such as NMN and NR has been a challenge due to the vulnerability of these compounds to enzymatic degradation, conversion in sample processing, and their complex behaviors in different column and extraction conditions. We have previously established a highly quantitative methodology to measure NAD^+^ and NMN levels in biological samples with high-performance liquid chromatography (HPLC) and have been using this method for mouse and human samples^14–17^. However, NMN levels are usually much lower than NAD^+^ levels, so the quantitation of NMN in biological samples has long been debated.

Several groups have been using their own methodologies with liquid chromatography-tandem mass spectrometry (LC-MS/MS) to detect and measure NMN in biological samples^18–22^. However, there has been no comprehensive assessment for extraction methods, recovery efficiencies, and the effects of the complex biochemical context of samples known as matrix effects. In this study, we assessed these issues and successfully developed a LC-MS/MS-driven methodology using double isotopic NMN standards (double isotope-mediated LC-MS/MS; dimeLC-MS/MS) for the accurate and reliable quantification of NMN in biological samples.

## Results

### Preparation of a new column and isotopic compounds

There are multiple steps that could cause inaccuracy of measurement for NMN and NR quantitation. For example, differences in sample collection and extraction methodologies significantly affect the measurement of NAD^+^ intermediates^23,24^. Whereas the C18 column allows us to accurately measure NAD^+^ levels in HPLC^15^, the porous graphitic carbon column or the hydrophilic column works much greater to detect and measure NMN^14,16^. In mass spectrometry, ionization efficiencies of NMN and its related metabolites are significantly different and depend on the biochemical context of the sample extracts, called the matrix effect. Therefore, to achieve accurate, quantitative measurements of NMN and its related metabolites, all of these issues needed to be improved.

**Fig. 1:**
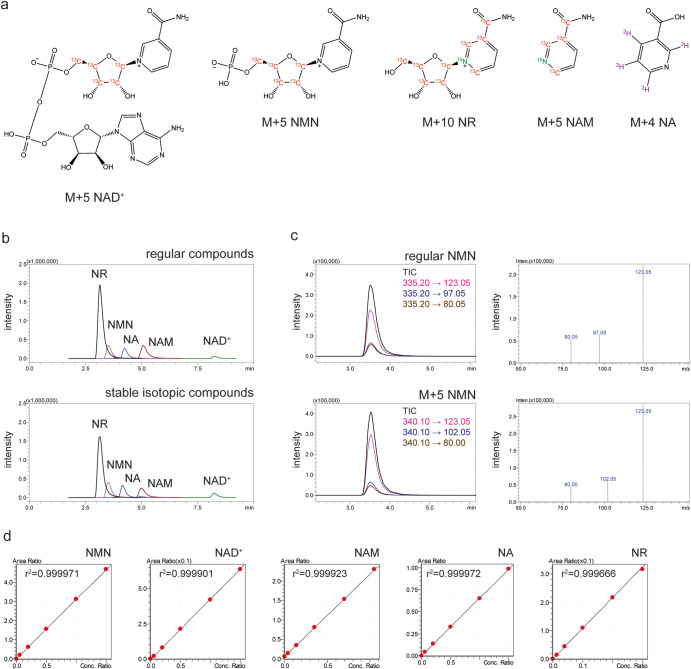
Isotopic compounds used for LC-MS/MS analysis. **a** The structures of stable isotopic compounds used for LC-MS/MS analysis. Atoms in red, green, or purple are stable isotopes. **b** Chromatograms of regular (upper panel) and stable isotopic (bottom panel) compounds. These regular and isotopic compounds (200 nM for NR and M + 10 NR; 1000 nM for NMN, NA, NAM, NAD^+^, M + 5 NMN, M + 4 NA, M + 5 NAM, and M + 5 NAD^+^) were separated by an NMN-2 column newly developed by Shimadzu Corporation. Total Ion Counts (TICs) from the multiple reaction monitoring (MRM) of each compound are shown. **c** Chromatograms and mass spectra of regular and isotopic NMN. TICs and transitions were obtained as a chromatogram from the MRM of regular and isotopic NMN (left panels), and resultant product ions were displayed as mass spectra (right panels). **d** Calibration curves of NMN and other related compounds. The area under curves (AUCs) of each regular compound at different ranges of concentrations (0, 60, 200, 500, 1000, and 1500 nM for NMN, NAD^+^, and NA; 0, 84, 280, 700, 1400, and 2100 nM for NAM; 0, 2.4, 8, 20, 40, and 60 nM for NR) were measured by LC-MS/MS under the existence of a fixed amount (1000 nM for M + 5 NMN, M + 5 NAD+ and M + 4 NA; 2000 nM for M + 5 NAM; 200 nM for M + 10 NR) of each corresponding isotopic compound and normalized to the AUC of each isotopic internal standard.

We first developed a new column that allows for stable and reproducible detection of NMN. This new column, Prototype NMN-2, contained C18-based high-purity silica particles to bind hydrophilic compounds more than carbon particles, improving the ability of separation, particularly for phosphate group-containing compounds, such as NMN and NAD^+^ (see details in the Materials and Methods section). We next synthesized stable isotopic compounds for NAD^+^ (M + 5), NMN (M + 5), NR (M + 10), NAM (M + 5) and NA (M + 4) to adjust the matrix effects of biological extracts (Fig. 1a). These isotopic compounds displayed identical retention times and relative mass differences to the regular compounds (Fig. 1b, c). After examining a linearity range of concentrations for each of regular and stable isotopic standard compounds (Supplementary Fig. 1), the standard curves of each regular compound normalized to each corresponding isotopic compound were drawn. They showed accurate linearity between 10 nM and 10 μM for NMN, NAM, NA, and NAD^+^ and between 0.04 nM and 400 nM for NR (Fig. 1d).

### Quantification of NMN in mouse plasma samples after NMN administration

**Fig. 2:**
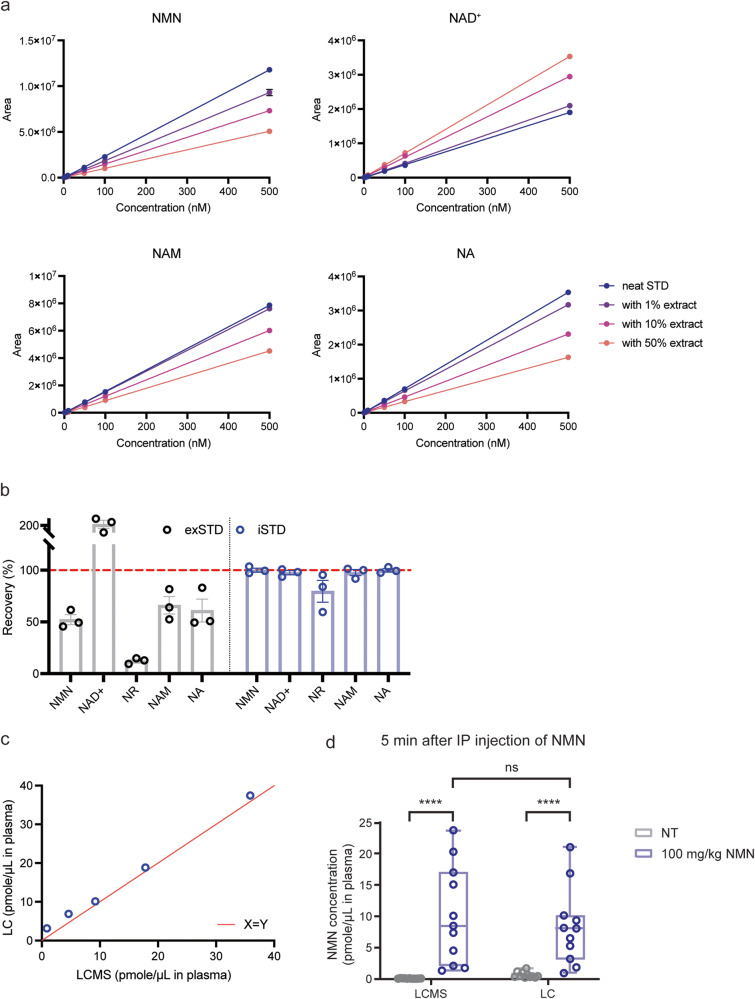
Adjustment of matrix effects and measurement of NMN in mouse plasma samples. **a** The matrix effects of mouse plasma on AUCs of NMN, NAD^+^, NAM, and NA. Zero, 50, 100 or 500 nM concentrations of NMN, NAD^+^, NAM, and NA standard solutions were combined with PCA extracts from mouse plasma at 1%, 10% or 50% concentrations. Error bars indicate SEM obtained from triplicate injections to LC-MS/MS. **b** Adjustment of matrix effects by an internal standard method using stable isotopic compounds. Regular compounds were added to mouse plasma at the final concentration of 1 μM. Recovery efficiencies were calculated by an external standardizing method (exSTD, gray bar) or an internal standardizing method (iSTD, blue bar). Means were calculated from three independent experiments. Data are presented as mean ± SEM. **c** Comparison of HPLC-based and LC-MS/MS-based methodologies for NMN measurement. Mouse plasma was spiked with indicated concentrations of NMN. The same extracts from NMN-spiked plasma were measured by LC-MS/MS (x axis) and HPLC (y axis). **d** NMN levels in mouse plasma after NMN IP injection. Three- to four-month-old C57BL/6 J mice (*n* = 10) were given NMN at 100 mg/kg by IP injection. Plasma was extracted with PCA, and NMN was measured by both HPLC-based and LC-MS/MS-based methodologies. NMN levels are shown in a box-and-whisker plot. The box extends from the 25th to 75th percentiles, and whiskers go down to the minimum and up to the maximum values. The center line indicates a median. Each individual value is also plotted on the graph. Statistical analysis was conducted using repeated-measures two-way ANOVA with Bonferroni’s multiple comparisons post hoc test. ****p < 0.0001.

When we developed HPLC-based methodologies for NAD^+^ and NMN measurements, we were aware that it was critical to use a very strong acid, perchloric acid (PCA), to efficiently extract NAD^+^ and NMN from biological samples such as plasma with minimal losses^14–17^. For mass spectrometry-based measurements, it is also necessary to be cognizant of and adjust for the matrix effect. Using regular compounds, we examined the matrix effect of mouse plasma extracted with PCA by adding the plasma extract at 1%, 10%, and 50% concentrations to varying concentrations of NMN, NAD^+^, NAM, and NA (Fig. 2a). Interestingly, the areas under the curves (AUCs) were increased for NAD^+^, whereas the AUCs were decreased for NMN. For example, 50% mouse plasma extract showed 185.6% increases in NAD^+^ AUCs at 500 nM, whereas the same concentration of mouse plasma extract suppressed NMN AUCs by 57.0% at 500 nM, suggesting that mouse plasma extract has opposite matrix effects for NAD^+^ and NMN. Similarly to NMN, mouse plasma extract suppressed AUCs for NAM and NA (Fig. 2a).

To adjust for such matrix effects, a fixed amount (1 μM) of each isotopic compound was added to mouse plasma samples prior to the PCA extraction. One μM of each regular compound was also spiked to the same plasma samples prior to the PCA extraction. After extraction, levels of each added regular compound were quantitated with standard curves using each regular compound (external standardizing method; exSTD) or after being normalized to each internal isotopic standard (1 μM) and compared to a standard curve that was also normalized to the internal isotopic standard (internal standardizing method; iSTD). Then, percentages of recovery were calculated (compared to original 1 μM of each regular compound added). When matrix effects are properly adjusted by normalizing to internal isotopic standards, the expected recovery percentages should be 100%. Recovery efficiencies of NMN, NAD^+^, NAM, and NA reflected varying matrix effects when values were measured by the exSTD (Fig. 2b, left), but as expected, they showed approximately 100% recovery after adjusting matrix effects by the iSTD (Fig. 2b, right). These results strongly suggest that the adjustment of each matrix effect by normalizing to each internal isotopic standard is critical for the accuracy of the measurement. Also worth noting is that NR showed 89.1% recovery after the adjustment, which significantly improved the accuracy of the measurement by the exSTD (Fig. 2b). This is because the PCA extraction affected the elution pattern of NR (Supplementary Fig. 2a) so that the ratio between regular NR and isotopic NR was altered. Currently, the reason why the elution pattern of NR was changed remains unclear. We also determined lowest concentrations that still allowed reliable recoveries for NMN, NAM, NA, and NAD^+^ by measuring recovery efficiencies with the iSTD. We found that stable recoveries were achieved down to 0.1, 0.07, 0.05, and 0.5 μM for NMN, NAM, NA, and NAD^+^, respectively (Supplementary Fig. 2b), which comprise detection limits for each compound.

We then measured mouse plasma samples spiked with different concentrations of NMN by using the mass spectrometry-based method with the iSTD adjustment of its matrix effect and the HPLC-based method that we previously developed^15^. Remarkably, values from both mass spectrometry-based and HPLC-based measurements were almost identical (Fig. 2c), confirming the importance of the PCA extraction and the accuracy of both methodologies. We also measured NMN concentrations in mouse plasma 5 min after intraperitoneal (IP) injection of NMN at the dose of 100 mg/kg. We compared NMN values measured by both methodologies. Interestingly, both values were very consistent after NMN administration, with concentrations being highly variable, ranging from 2–3 μM to 20 μM, depending on individual mice (Fig. 2d). The levels of increases in plasma NMN concentrations after NMN IP administration were also very similar between these two methodologies (Supplementary Fig. 2c). These results nicely confirm a very fast kinetics of NMN uptake into blood circulation, as originally reported^14,16,24^. It should be noted that HPLC- and mass spectrometry-based methodologies gave very different values for endogenous NMN levels in mouse plasma (before NMN administration). The HPLC-based measurement gave much higher values of endogenous NMN in mouse plasma compared to those by mass spectrometry. Although the reason for this discrepancy is currently under investigation, we will come back to this particular issue in the Discussion section.

### Absolute quantitation of NMN levels in biological samples

**Fig. 3:**
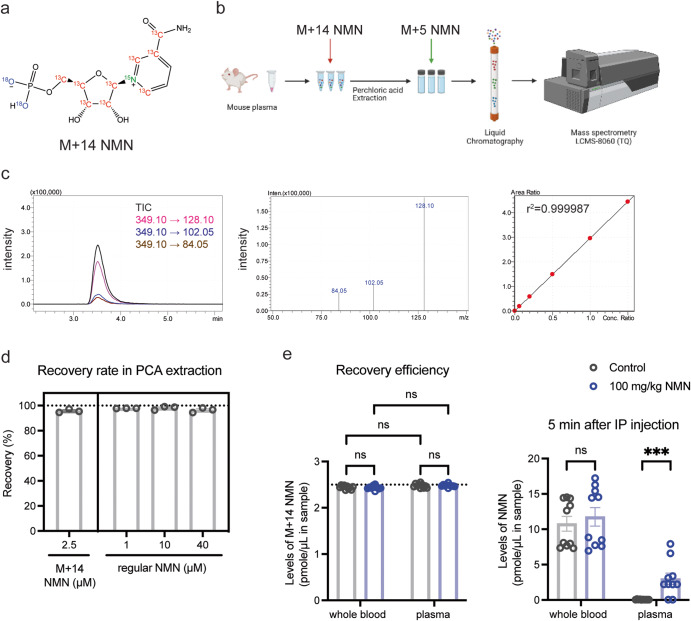
The double isotope-mediated LC-MS/MS methodology (dimeLC-MS/MS) for NMN measurement. **a** The structure of NMN (M + 14) with stable isotopes. Isotopic carbon, nitrogen, and oxygen are shown in red, green, and purple colors, respectively. **b** The schematic flow of the dimeLC-MS/MS for NMN measurement. M + 14 NMN is added directly to the mouse plasma before extraction by perchloric acid. M + 5 NMN is added to the final, neutralized extract before detection by LC-MS/MS. **c** The chromatogram, mass spectra, and calibration curve of NMN (M + 14). TICs and transitions were obtained from the MRM of 1000 nM NMN (M + 14) and plotted in a chromatogram (left panel), and corresponding product ions were displayed as mass spectra (middle panel). The calibration curve of NMN (M + 14) was drawn at the range of 0, 60, 200, 500, 1000, and 1500 nM after normalizing its AUCs to those of 1000 nM NMN (M + 5). **d** Recovery efficiencies of spiked NMN (M + 14) and regular NMN in the dimeLC-MS/MS. NMN (M + 14) and indicated concentrations of regular NMN were added to mouse plasma. Each plasma sample was extracted by PCA and analyzed with dimeLC-MS/MS. Recovery efficiencies were calculated by an iSTD method. Results were obtained from three independent experiments. **e** NMN levels in mouse whole blood or plasma after NMN IP injection. Three- to four-month-old C57BL/6 J mice (*n* = 10) were given NMN by IP injection (100 mg/kg). Each sample was extracted by PCA, and NMN was measured by dimeLC-MS/MS. Repeated-measures two-way ANOVA was conducted to compare the results with Bonferroni’s multiple comparisons *post hoc* test. Data are presented as mean ± SEM. ***p < 0.005.

The fact that the recovery efficiency is approximately 100% for NMN (Fig. 2b) strongly suggests that NMN is not degraded or altered to other related compounds such as NAM and NR in this particular procedure of sample collection and extraction^20,23,24^. However, in general, NMN is susceptible for enzymatic degradation or conversion during sample collection and extraction. Therefore, it is critical to be able to monitor and control such processes of NMN degradation and conversion throughout sample collection and extraction. Therefore, we decided to use another isotopic NMN, namely NMN (M + 14), to monitor those processes (Fig. 3a). By adding NMN (M + 14) to biological samples such as plasma immediately after their collection, we can monitor the fate of NMN during the entire process of sample handling (Fig. 3b). We also added NMN (M + 5) after PCA extraction to adjust the matrix effect (Fig. 3b). NMN (M + 14) showed the identical retention time and relative mass difference to regular NMN, and accurate linearity in its measurement (Fig. 3c). By using these two different isotopic NMN compounds and assessing the ratio of NMN (M + 14) and NMN (M + 5), the exact concentration of NMN (M + 14) can be calculated, which allows us to calculate the exact recovery efficiency in the PCA extraction. To prove this, 2.5 μM of NMN (M + 14) was added to mouse plasma prior to the PCA extraction, and after extraction 500 nM of NMN (M + 5) was added to adjust the matrix effect (Fig. 3d). We then examined the recovery efficiency of 2.5 μM NMN (M + 14) and regular NMN at 1, 10, and 40 μM in the PCA extraction. Again, the recovery efficiency was almost ~100% (95.3%–99.1%) for both NMN (M + 14) and regular NMN (Fig. 3d), which definitively confirms the importance of the PCA extraction.

To further demonstrate the advantage of this double-isotope methodology, we measured NMN concentrations in mouse whole blood and plasma; two different biological samples which may have different matrix effects. In both samples, NMN concentrations were quantitated 5 min after IP injection of NMN at a dose of 100 mg/kg. 2.5 μM of NMN (M + 14) was added into mouse whole blood or plasma prior to PCA extraction, and 500 nM of NMN (M + 5) was added to each extract. Again, after adjusting matrix effects of whole blood and plasma, the extraction efficiencies of NMN (M + 14) were close to 100% for both whole blood and plasma samples from control and NMN-administered mice (Fig. 3e, left). Whereas NMN concentrations in whole blood extracts did not significantly differ between control and NMN-administered mice, NMN concentrations in plasma extracts showed significant increases in NMN-administered mice (Fig. 3e, right). These results strongly suggest that although NMN degradation and conversion were minimal during the sample processing of both whole blood and plasma, plasma samples, which were immediately separated after blood collection, should be used to clearly detect NMN increases after NMN administration. Taken together, this new methodology with double isotopic NMN standards proves its great advantage to accurately monitor the fate of NMN and evaluate the extraction efficiency and the absolute concentrations of NMN in different types of biological samples.

### Comparison of PCA and MeOH extraction methods

**Fig. 4:**
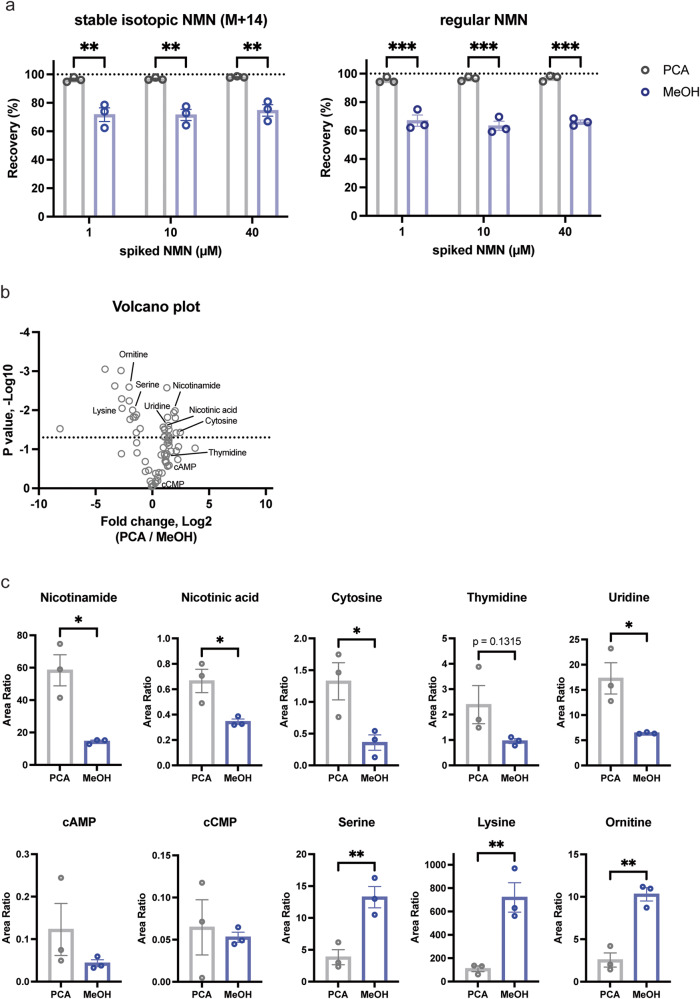
Comparison of PCA and MeOH extraction methods for NMN and other metabolites. **a** Recovery efficiencies of spiked NMN (M + 14) and regular NMN after PCA and MeOH-chloroform extraction. Indicated concentrations of NMN (M + 14) and regular NMN were added to mouse plasma. After extracting plasma samples with PCA or MeOH-chloroform, each extract was analyzed with dimeLC-MS/MS. Recovery efficiencies were calculated by an iSTD method. Means were obtained from three independent experiments. Two-way ANOVA was conducted to compare the results with Bonferroni’s multiple comparisons post hoc test. Data are presented as mean ± SEM. **p < 0.005; ***p < 0.001. **b** Comparison of PCA and MeOH extraction methods for primary metabolites in plasma. Plasma samples were extracted by PCA or MeOH-chloroform extraction, and 96 primary metabolites were analyzed using the Primary Metabolites Method Package Ver. 2. Average area ratios from three independent experiments were plotted in a volcano plot. **c** Area ratios of 10 representative compounds are shown for PCA and MeOH-chloroform extraction methods. Unpaired Student’s *t* test was conducted to compare the results. Data are presented as mean ± SEM. *p < 0.05; **p < 0.01.

Mass spectrometry-driven metabolomic analysis usually uses methanol (MeOH) to extract as many metabolites as possible. However, it remains unclear whether this MeOH-based extraction is suitable for the measurement of NMN and its related metabolites. Using this double isotope-mediated LC-MS/MS methodology (dimeLC-MS/MS), we compared the recovery efficiencies between PCA and MeOH. Whereas we again confirmed nearly 100% recovery efficiency in PCA extraction, MeOH extraction showed ~70% recovery efficiency for both NMN (M + 14) and regular NMN at 1, 10, and 40 μM (Fig. 4a), suggesting that a MeOH-based extraction method is not suitable for accurate NMN measurement. We also compared recovery efficiencies for NAM, NA, NAD^+^, and NR at different concentrations between PCA and MeOH extraction (Supplementary Fig. 3). In all cases, PCA extraction was significantly better than MeOH extraction.

We next examined whether such differences could be observed for other metabolites between PCA and MeOH extraction methods. For this assessment, mouse plasma samples were extracted with PCA or MeOH-chloroform with spiking of 100 μM 2-morpholinoethanesulfonic acid (MES) as an internal standard. For metabolite analysis, Primary Metabolites Method Package Ver. 2, which can detect and analyze 96 metabolites, was used (see the Materials and Methods section). Interestingly, metabolites were divided into two groups: one group of metabolites extracted better by PCA and the other extracted better by MeOH (Fig. 4b). NAM, NA, nucleosides (cytosine, thymidine, and uridine), and cyclic nucleotides (cAMP and cCMP) belong to the PCA group, whereas amino acids (serine and lysine) and ornithine belong to the MeOH group (Fig. 4c). These results strongly suggest that it is critical to employ the PCA extraction method, not a MeOH-based extraction method, to perform an accurate absolute quantification of precursors and intermediates for NAD^+^ in biological samples.

### Quantitative measurement of direct incorporation of NMN into cells

**Fig. 5:**
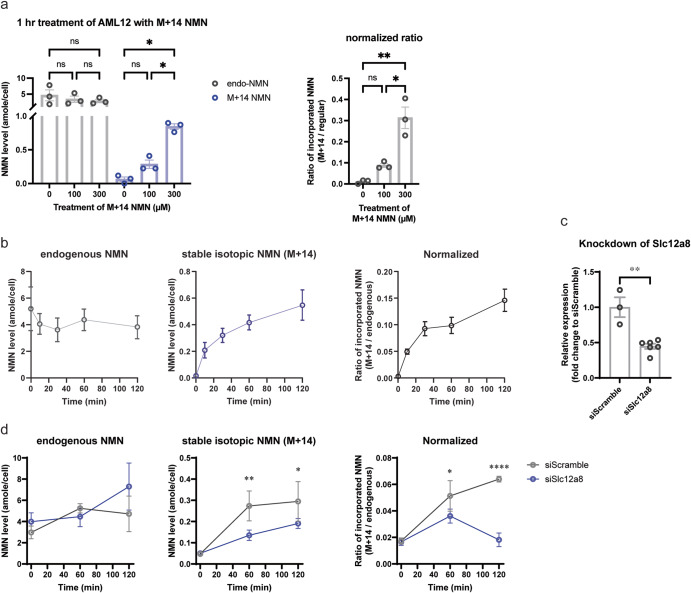
Accurate quantitation of NMN uptake into AML12 cells. **a** Dose-dependent increases of NMN (M + 14) uptake into AML12 cells 1 h after NMN addition to the media. Indicated concentrations of NMN (M + 14) were added to the culture media of AML12 cells. One hour after NMN addition, absolute amounts of regular NMN (left panel, gray bar), which represents an endogenous NMN pool, and NMN (M + 14) (left panel, blue bar), which represents NMN directly transported into AML12 cells, were quantitated by dimeLC-MS/MS. Ratios of the transported NMN (M + 14) to the endogenous NMN pool (regular NMN) were calculated (right panel). Results were obtained from three independent experiments. One-way ANOVA was conducted with Bonferroni’s multiple comparisons post hoc test. Data are presented as mean ± SEM. *p < 0.05; **p < 0.01. **b** The time course of NMN uptake into AML12 cells. AML12 cells were treated with 200 μM NMN (M + 14) over 2 h. Absolute amounts of regular NMN (right graph) and NMN (N + 14) (middle graph) were quantitated by dimeLC-MS/MS at 10, 30, 60, and 120 min time points after the addition of NMN (M + 14). Results were obtained from three independent experiments. Data are presented as mean ± SEM. **c** Knockdown efficiencies for *Slc12a8* mRNA expression in AML12 cells. Scrambled siRNA (siScramble) and siRNAs against Slc12a8 (siSlc12a8) were transfected to AML12 cells. The *Slc12a8* mRNA expression levels in knockdown cells were normalized to those in siScramble-transfected control cells. Two-tailed unpaired Student’s *t* test was conducted to compare the results. Data are presented as mean ± SEM. **p = 0.0013. **d** The uptake of NMN (M + 14) in *Slc12a8*-knockdown (*Slc12a8*-KD) AML12 cells. siScramble or siSlc12a8-transfected AML12 cells were treated with 200 μM NMN (M + 14). Absolute amounts of endogenous NMN (left graph) and NMN (M + 14) (middle graph) were quantitated by dimeLC-MS/MS at 0, 60, and 120 min time points after the addition of NMN (M + 14). The ratios of incorporated to endogenous NMN were also calculated (right graph). Results were obtained from two independent experiments with triplicate samples. Data are presented as mean ± SEM. Two-way ANOVA was conducted to compare the results with Bonferroni’s multiple comparisons *post hoc* test. *p < 0.05, **p < 0.005, ****p < 0.0001.

We next employed the dimeLC-MS/MS methodology to quantitatively measure the direct incorporation of NMN into cultured cells. We chose a mouse hepatocyte cell line AML12 because it is reported that AML12 cells show a relatively high expression of Slc12a8, a newly identified NMN transporter^18^. We also used 78c (CD38 inhibitor), adenosine-5’-(α,β-methylene) diphosphate (AOPCP, CD73 inhibitor), dipyridamole (ENT inhibitor), and FK-866 (NAMPT inhibitor) to avoid the degradation of NMN in the culture media and suppress possible contribution of NAM and NR to the endogenous NMN pool^18^. We added NMN (M + 14) into culture media at the final concentration of 100 or 300 μM and then measured and calculated the absolute levels of regular NMN and NMN (M + 14) as a unit of atto mole (10^−18^ mole; amole)/cell at 1 h time point after NMN addition. During calculation, all measured AUCs of regular NMN and NMN (M + 14) were normalized to those of NMN (M + 5). Interestingly, an endogenous NMN pool (regular NMN) was around 5 amole/cell, and it was not significantly affected by treating cells with 100 or 300 μM of NMN (M + 14) for 1 h (Fig. 5a, left). However, a dose-dependent direct uptake of NMN (M + 14) was clearly detected (Fig. 5a, left), and the extent of this direct uptake was up to 30% of the endogenous NMN pool (Fig. 5a, right). We then examined the time course of the NMN uptake by adding 200 μM of NMN (M + 14) into culture media. NMN was quickly transported into cells at 10 min time point, and the NMN levels increased steadily over 2 h, up to ~15% of the endogenous NMN pool (Fig. 5b). Meanwhile, the endogenous NMN pool did not change much, implicating an immediate utilization of NMN inside of cells (Fig. 5b). Indeed, NAD^+^ (m/z = 678) produced directly from NMN (M + 14) increased ~2-fold over 2 h (Supplementary Fig. 4a). NAD^+^ (m/z = 669) produced from NAM (M + 5), a degradation product of NMN (M + 14), also increased ~2-fold, suggesting that degradation of NMN still happened to some extent in this culture condition (Supplementary Fig. 4b).

To confirm that this uptake of NMN is dependent on *Slc12a8*, we knocked down *Slc12a8* in AML12 cells and examined NMN uptake in these *Slc12a8*-knockdown (*Slc12a8*-KD) cells. The knockdown efficiency was 55% (Fig. 5c). Interestingly, *Slc12a8*-KD AML12 cells showed significant decreases (49% and 65% of siScramble controls at 60 and 120 min time points) in the uptake of NMN (M + 14) (Fig. 5d), strongly suggesting that this NMN uptake is dependent on the function of the Slc12a8 NMN transporter. It should be noted that endogenous NMN levels moderately increased in *Slc12a8*-KD AML12 cells at 120 min time point, making the normalized NMN uptake significantly lower than control (Fig. 5d). These results clearly demonstrate that the dimeLC-MS/MS methodology allows us to accurately and quantitatively measure NMN uptake and also confirms that NMN can be transported directly into cells and utilized for NAD^+^ biosynthesis^24^.

### Comparison of plasma NMN, NAM, NA, and NR levels after oral gavage of NMN between young and old mice

**Fig. 6:**
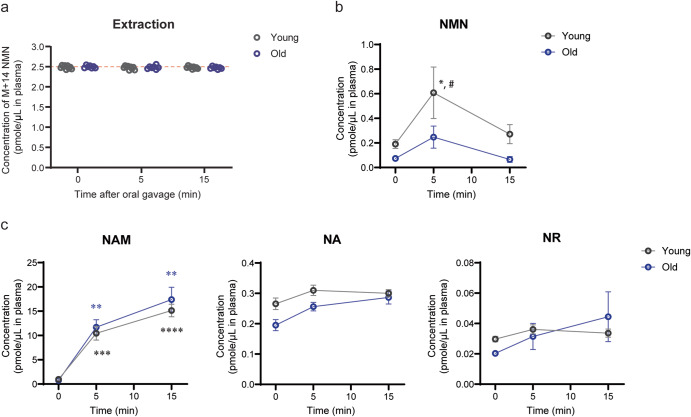
NMN increase in mouse plasma after oral gavage of NMN. Five- to six month-old (young, *n* = 9) or 24 to 25 month-old (old, *n* = 7) C57BL/6 J male mice were orally given NMN at 300 mg/kg. Blood was collected from the tail vein at indicated time points. Plasma was extracted with PCA and analyzed with dimeLC-MS/MS. Data are presented as mean ± SEM. Statistical analysis was conducted using repeated-measures two-way ANOVA with Bonferroni’s multiple comparisons post hoc test. **a** Recovery efficiencies for NMN (M + 14) in young and old plasma extracts are shown. **b** Changes in plasma NMN concentrations after oral gavage in young and old mice. *p < 0.05 between young mice and old mice at 5 min time point. #*p* < 0.05 between 0 and 5 min time points in young mice. **c** Changes in plasma NAM, NA and NR concentrations in young and old mice. Statistical significances at 5 and 15 min time points are shown against 0 min time point for NAM. **p < 0.005, ***p < 0.0005, ****p < 0.0001.

Lastly, we conducted oral gavage of NMN in 4–6 month-old (young) and 24–26 month-old (old) healthy wild-type B6 mice and measured plasma levels of NMN. The dose of NMN was 300 mg/kg, relatively a high dose for mice^14^. Again, extraction efficiencies for NMN were almost 100% (Fig. 6a). Plasma NMN levels significantly increased from 0.2 μM to 0.6 μM within 5 min and went back to basal levels by 15 min in young mice (Fig. 6b). Although old mice also showed increases in plasma NMN levels after oral gavage, the peak NMN levels at 5 min time point were significantly lower in old mice, compared to young mice. These results definitively confirm the very fast transfer of NMN to blood circulation, as we reported previously^14,24^. However, whereas the observed time courses and changes in plasma NMN levels in young and old mice were highly consistent with our previous results^14,24^, the absolute values of plasma NMN levels measured by the dimeLC-MS/MS methodology were much lower than those measured by the HPLC-based methodology. Given that NMN IP administration provided very consistent, high NMN values (around 10 μM as an average) in plasma at 5 min between both methodologies, this stark discordance between two methodologies was surprising. Thus, we decided to measure other metabolites, such as NAM, NA, and NR, in plasma after oral gavage of NMN. Whereas plasma NAM levels exhibited dramatic increases within 5–15 min in both young and old mice, NA and NR did not show any significant increases within 15 min in both young and old mice (Fig. 6c), strongly suggesting that a major degradation product of NMN in the gut is NAM. NAM is reportedly converted to NA by microbiome in 2–4 h^25^, but this did not happen within the first 15 min of NMN uptake. Given that plasma NAM levels were very similar between young and old mice after oral gavage of NMN and also that NAM, NA, and NR are clearly separable from NMN in the HPLC-based methodology, these metabolites cannot explain stark differences in absolute NMN values between two methodologies. Considering that such differences were observed only in oral gavage, microbiome may convert NMN to another unknown compound that cannot be distinguished by the HPLC-based methodology.

## Discussion

Quantitative measurement of NAD^+^ intermediates, NMN in particular, has long been a serious challenge in the field of NAD^+^ biology. One reason is that NMN can be easily degraded or converted to other related metabolites, such as NAM and NR, when collecting and processing biological samples. Blood is probably the most difficult biological sample to handle because blood carries significant activities of CD38 and CD73 (ecto-5’-nucleotidase), both of which could use NMN as a substrate^18,26^. Another reason is that the behavior of NMN in the column is very complex probably because of the bipartite nature of its charges so that subtle differences in extraction and column conditions significantly affect the reliable and accurate detection of NMN^24,27^. To overcome these problems, we have previously developed a HPLC-driven methodology to measure NMN levels in biological samples^14,16^. We employed this method to assess the pharmacokinetics of NMN after oral administration in mice and demonstrated that NMN uptake from the gut into blood circulation happened within 2–3 min and transported into tissues within 10–30 min^14^. This surprising finding led us to the discovery of a novel NMN transporter Slc12a8^24^. However, the difficulty with accurate NMN measurements has caused intense debates on whether NMN could be transported directly into cells or blood circulation^24,27,28^. In the present study, we were able to overcome these difficulties and successfully developed an accurate and reliable LC-MS/MS methodology using double isotopic NMN standards (dimeLC-MS/MS).

The advantage of the dimeLC-MS/MS is two-fold: First, we were able to confirm that immediate PCA extraction gave almost 100% recovery efficiency for NMN and other NAD^+^-related metabolites, compared to ~70% recovery efficiency by the MeOH extraction. Therefore, in this method, we were able to adjust matrix effects by adding an isotopic NMN standard, namely NMN (M + 5), to biological samples even after extraction. Adjusting the matrix effect is critical because the NMN AUCs were suppressed down to ~40% in mouse plasma extracts. The dimeLC-MS/MS method allows us to properly adjust matrix effects and quantitate NMN levels contained in extracts of biological samples. Second, adding the second isotopic standard, NMN (M + 14), to biological samples immediately after sample collection allowed us to trace the fate of NMN precisely during sample processing. Furthermore, by calculating the ratio of NMN (M + 14) and NMN (M + 5), the exact concentration of NMN (M + 14) can be calculated, which allows for calculation of the exact recovery efficiency in any extraction method. Therefore, the use of double isotopic NMN standards, NMN (M + 14) and NMN (M + 5), in the LC-MS/MS-driven methodology significantly increases the accuracy and the reliability of NMN measurement in biological samples.

By using this new methodology, we obtained three important biological results in this study. First, we were able to detect significant increases in plasma NMN levels 5 min after IP injection of NMN, whereas no increases in NMN levels were detected in whole blood extracts. Therefore, it is critical to assess plasma NMN levels, not whole blood NMN levels, and it is clear that NMN can be quickly transported to systemic circulation. Importantly, after NMN IP administration, the HPLC-driven methodology that we previously developed gave highly consistent values of plasma NMN concentrations with those by the LC-MS/MS-driven methodology after properly adjusting its matrix effect, providing convincing confirmation of the results by two independent methodologies. Both methodologies clearly demonstrated that plasma NMN concentrations reached quite high levels 5 min after IP administration.

Second, we were also able to detect increases in plasma NMN levels 5 min after oral gavage of NMN at the dose of 300 mg/kg in both young and old mice. Interestingly, the peak NMN levels at 5 min time point were significantly lower in old mice, compared to those in young mice, clearly confirming the rapid transfer of NMN into blood circulation and the age-associated decrease in the capability of NMN uptake^14,24^. It has been speculated that NMN would be converted to NR by CD73 in the gut^29^. However, at least within the time frame when NMN was rapidly transported to blood circulation, there was no significant conversion from NMN to NR. Yet, dramatic increases in plasma NAM levels were observed quickly after oral gavage, indicating that NMN is degraded to NAM in the gut. These findings emphasize the importance of examining earlier time points, at least minute-order, instead of hourly-order, to accurately detect the uptake of NMN and NMN-dependent NAD^+^ biosynthesis in vivo. An important remaining question is why the HPLC-driven and LC-MS/MS-driven methodologies give completely different values of plasma NMN after oral gavage and even for the basal levels, whereas both methodologies give highly consistent values after IP administration. Although the exact reason is currently under investigation, one interesting possibility would be that microbiome converts NMN quickly to another compound that is chemically very close to NMN so that the HPLC-driven methodology cannot distinguish the difference. The existence of such compound could explain the difference in detectable NMN levels between IP administration and oral gavage.

Lastly, by using this dimeLC-MS/MS, we were able to show that NMN was transported directly into AML12 cells within 10 min and also that levels of transported NMN increased up to ~30% of the endogenous NMN pool, clearly demonstrating that a significant amount of NMN can be transported directly into cells. Similar results were obtained previously in primary hepatocytes, and this direct transport of NMN was almost completely abrogated in *Slc12a8*-deficient hepatocytes^24^. Consistent with these results, knocking down *Slc12a8* in AML12 cells also decreased the uptake of NMN significantly. Whereas we were not able to calculate the absolute amounts of transported NMN in our previous study^24^, we were able to accurately quantitate the absolute levels of the endogenous NMN pool and the NMN transported into cells in the present study. An interesting observation is that in *Slc12a8*-KD AML12 cells, endogenous NMN levels moderately increased, which makes the relative NMN uptake much lower than control. Given that the endogenous NMN pool did not change much even when NMN was transported up to 30% of its endogenous pool, these findings may implicate some strict feedback mechanism between the Slc12a8-dependent NMN uptake and the maintenance of endogenous NMN pool. Indeed, when NMN (M + 14) was transported into cells, NAD^+^ directly synthesized from NMN (M + 14) (m/z = 678) significantly increased over 2 h. Thus, when NMN uptake was reduced, we speculate that some feedback mechanism may try to maintain the endogenous NMN pool. Taken together, the dimeLC-MS/MS will be powerful to examine the precise kinetics and the fates of NMN and NAD^+^-related metabolites in cells or tissues.

In conclusion, we successfully developed a new LC-MS/MS-driven methodology with double isotopic NMN standards, named dimeLC-MS/MS, and demonstrated its accuracy and reliability to measure NMN in biological samples. The dimeLC-MS/MS allowed us to accurately measure plasma NMN levels after IP injection and oral gavage in mice and also conduct absolute quantitation of NMN amounts directly transported into cells. The dimeLC-MS/MS method will open many interesting opportunities to assess the kinetics of NMN uptake and NAD^+^ biosynthesis in different metabolic conditions and tissues.

## Methods

### Chemicals and reagents

NMN was a gift from Mirai Lab Biosciences Ltd. (Tokyo, Japan). NAD^+^ (#N1511), NR (#SMB00907), NAM (#72340), and NA (#N0761) were purchased from Sigma (USA). [^13^C_5_]-NMN (#C7934), [^13^C_9_, ^15^N, ^18^O_2_]-NMN (#C7991), [^13^C_9_, ^15^N]-NR (#C7990), and [^2^H_4_]-NA (#C2885) were purchased from Alsachim (France). Other isotopic compounds were custom-synthesized in Alsachim.

### Mouse experiments

All animal studies were approved by the Institutional Animal Care and Use Committee of Washington University in St. Louis, Missouri, USA (Protocol No. 22-0007), and were in accordance with the National Institutes of Health guidelines. C57BL/6 J mice were group-housed in our SPF mouse facility with 12-h light/12-h dark cycles. All mice received a regular chow diet *ad libitum* (PicoLab 5053 Rodent Diet 20; Lab Diets). Blood samples were collected from the tail vein with microhematocrit heparinized capillary tubes (Fisher Scientific), and plasma was immediately separated after blood collection. NMN administration was carried out by intraperitoneal (IP) injection or oral gavage, as described previously^14,16,24^. The mice used in this study were not sacrificed and are still alive or being used for other training purposes.

### Perchloric acid (PCA) extraction of mouse plasma samples

Plasma was separated from mouse whole blood by centrifugation at 3873 × *g* at 4 °C for 7 min in a micro-centrifuge tube. Mouse plasma was then mixed with an ice-cold 10% PCA solution containing stable isotopic compounds as internal standards and then incubated on ice for 15 min. After centrifugation at 21,500 × *g* at 4 °C for 5 min, the supernatant was separated and neutralized with ice-cold 3 M K_2_CO_3_ solution at a 1:3 ratio. Then, the mixture was incubated on ice for 15 min with a lid opened. The resulting salt was pelleted by centrifugation at 21,500 × *g* at 4 °C for 5 min. The resulting supernatant (final extract) was measured with the High Performance Liquid Chromatography Triple Quadrupole Mass Spectrometer system (LC-MS/MS, LCMS-8060, Shimadzu). Concentrations of NMN and other related metabolites in extracts were quantitated based on each area under curve (AUC) compared to a standard curve (normalized by a standard isotopic compound) and back calculated by normalization of dilution rate and plasma volume.

### MeOH-chloroform extraction

200 μL of MeOH containing an internal standard was added to 20 μL of mouse plasma and mixed well for few minutes. Then, 200 μL of chloroform was added and mixed with a vortexer. 80 μL of LCMS-grade water was added and mixed well. The mixture was centrifuged at 21,500 × *g* at 4 °C for 15 min. 200 μL of an upper water phase was transferred to a new tube, then dried up using speed-vac. Metabolites were dissolved with 100 μL of LCMS-grade water and measured with LC-MS/MS (LCMS-8060, Shimadzu).

### LC-MS/MS analysis

LC-MS/MS analysis was conducted using a Nexera X2 Ultra HPLC system coupled to a triple quadrupole mass spectrometer (LCMS-8060, Shimadzu). Prototype Column NMN-2 (150 mm × 2.0 mm, 2.2 µm particle size, Shimadzu) was developed specifically for the purpose of this research. Prototype Column NMN-2 contained the C18-based high-purity silica particles to bind hydrophilic compounds more than carbon particles and to avoid small debris. The particle size was specifically designed to be 2.2 μm to confer clear separation. The diameter of the column was also specifically designed to have 2 mm to achieve high sensitivity. Non-metal materials were used for all wetted parts to avoid metal chelate adsorption. With these improvements, Prototype Column NMN-2 can be easily cleaned with organic solvents.

Samples were kept in an autosampler at 4 °C during analysis, and 2 µL of each sample was injected to Prototype Column NMN-2. Column temperature was kept at 21 °C. Mobile phase consisted of water (A) and acetonitrile (B), both containing 0.1% formic acid. Using a flow rate of 0.2 mL/min, chromatographic separation was achieved with the gradient elution time program as follows: 1% B (0–2 min), 1–38.6% B (2–10 min), 95% B (10.01–12 min), and 1% B (12.01–15 min). NMN and NAD^+^ were typically eluted at 3.5 min and 7.5 min, respectively.

Metabolites were detected by single reaction monitoring, also known as multiple reaction monitoring (MRM), in the positive mode after electrospray ionization (ESI). The target cycle time was set at 0.4 s with a 1 ms pause between each transition. The dwell time for each transition was automatically set at 10–15 ms. Other parameters for the mass spectrometer were set as follows: interface voltage of 3.5 kV, nebulizing gas flow of 3 L/min, heating gas flow of 10 L/min, drying gas flow of 10 L/min, interface temperature of 300 °C, desolvation line temperature of 200 °C, heat block temperature of 350 °C, and collision-induced dissociation gas pressure of 270 kPa. All data were processed by LabSolutions software (Shimadzu).

### Metabolomics analysis

Metabolites were extracted with PCA extraction or MeOH-chloroform with the addition of 100 μM 2-morpholinoethanesulfonic acid (MES) as an internal standard. MES has a good solubility in water and is also resistant to acids, bases, and light, providing a minimal loss in extraction and stable and reliable results in different experimental conditions. Metabolites were analyzed by Primary Metabolites Method Package ver.2 (Shimadzu) with LC-MS/MS (LCMS-8060, Shimadzu). Each area ratio was obtained by dividing the AUC of a target metabolite with the AUC of the internal standard.

### Cell culture and drug treatment

AML12 cells were obtained from the American Type Culture Collection (ATCC) and maintained in DMEM/F12 medium supplemented with 10% FBS, 1% penicillin–streptomycin (Life Technologies), 40 ng/mL dexamethasone, 0.005 mg/mL insulin, 0.005 mg/mL transferrin and 5 μg/mL selenium (Insulin-Transferrin-Selenium [ITS-G], #41400045, Gibco). Dexamethasone and ITS were removed from the AML12 medium during drug treatments. In NMN uptake experiments, 8 × 10^5^ cells of AML12 were plated in the 6 cm dish and treated with 0.5 μM 78c (CD38 inhibitor) and 50 μM adenosine-5’-(α,β-methylene) diphosphate (AOPCP, CD73 inhibitor) for 16 h. Then, 2 μM dipyridamole (ENT inhibitor) and 100 nM FK-866 (NAMPT inhibitor) were added to the media for 1 h. After treating cells with all inhibitors, indicated concentrations of stable isotopic NMN (M + 14) were added to AML12 cells without removing inhibitors. After washing with PBS at least three times, cells were harvested with trypsin-EDTA solution and frozen at −30 °C until analysis. For measurement of NMN incorporated into the cell, cell pellets were resuspended in LC/MS-grade water containing stable isotopic NMN (M + 5) and then extracted with perchloric acid, as described previously^24^. For knockdown of *Slc12a8* in AML12 cells, scrambled siRNA and siRNAs against *Slc12a8* (#10; CCUACAUCAUGGUGGACUA, #11; GCAGGUGUCAAGUGGAUUA, Dharmacon, Horizon Discovery, Ltd.) were transfected to AML12 cells using siQUEST (Mirus Bio LLC, WI). After 24 h of transfection, both scrambled siRNA- and *Slc12a8* siRNA-transfected cells (siScramble and si*Slc12a8*, respectively) were washed with warmed PBS(-), and then the medium was added to the transfected cells. Inhibitors treatment, 200 μM NMN (M + 14) treatment, cell harvest, and analysis were done as described above.

### Statistical analysis

All data are presented as mean or mean ± SEM. Statistical significance between control and experimental samples was determined by unpaired Student’s *t* test. Statistical significance for differences in multiple NMN samples was analyzed by one-way, two-way, or repeated-measures two-way ANOVA with Bonferroni’s multiple comparisons test as a post hoc test. *p* values < 0.05 were considered statistically significant. All statistical tests were performed using GraphPad Prism (Ver 10.1.0).

### Reporting summary

Further information on research design is available in the Nature Research Reporting Summary linked to this article.

### Supplementary information

Supplementary Information

Supplementary table

Reporting Summary

## Data Availability

All data generated or analyzed during this study are included in this manuscript. Any additional materials are available from the corresponding author on reasonable request.
