# Supplementary material for: Absolute quantification of nicotinamide mononucleotide in biological samples by double isotope-mediated liquid chromatography-tandem mass spectrometry (dimeLC-MS/MS)
Source: NPJ Aging. 2024 Jan 2;10(1):2. doi: 10.1038/s41514-023-00133-1 (PMC10762063; doi:10.1038/s41514-023-00133-1)
Supplement: Supplementary file 1 — Supplementary Information [file 41514_2023_133_MOESM1_ESM.pdf]

Supplementary Information

Absolute quantification of nicotinamide mononucleotide in biological samples by a double isotope-mediated liquid chromatography-tandem mass spectrometry method (dimeLC-MS/MS)

Junya Unno[1,4], Kathryn F. Mills[4], Tairo Ogura[2], Masayuki Nishimura[3], Shin-ichiro Imai[4]

[1] Technology Research Laboratory, Shimadzu Corporation, Kyoto, Japan

[2] Innovation Center, Shimadzu Scientific Instruments, Inc., Columbia, Maryland, USA

[3] New Strategy Department, Shimadzu Scientific Instruments, Inc., Columbia, Maryland, USA

[4] Department of Developmental Biology, Washington University School of Medicine, St. Louis, Missouri, USA

Correspondence:
Shin-ichiro Imai, M.D., Ph.D.
Theodore and Bertha Bryan Distinguished Professor
in Environmental Medicine
Department of Developmental Biology, Department of Medicine (Joint)
Washington University School of Medicine in St. Louis
Phone (Office): 314-362-7228
E-mail: imaishin@wustl.edu

Supplementary Figure 1:

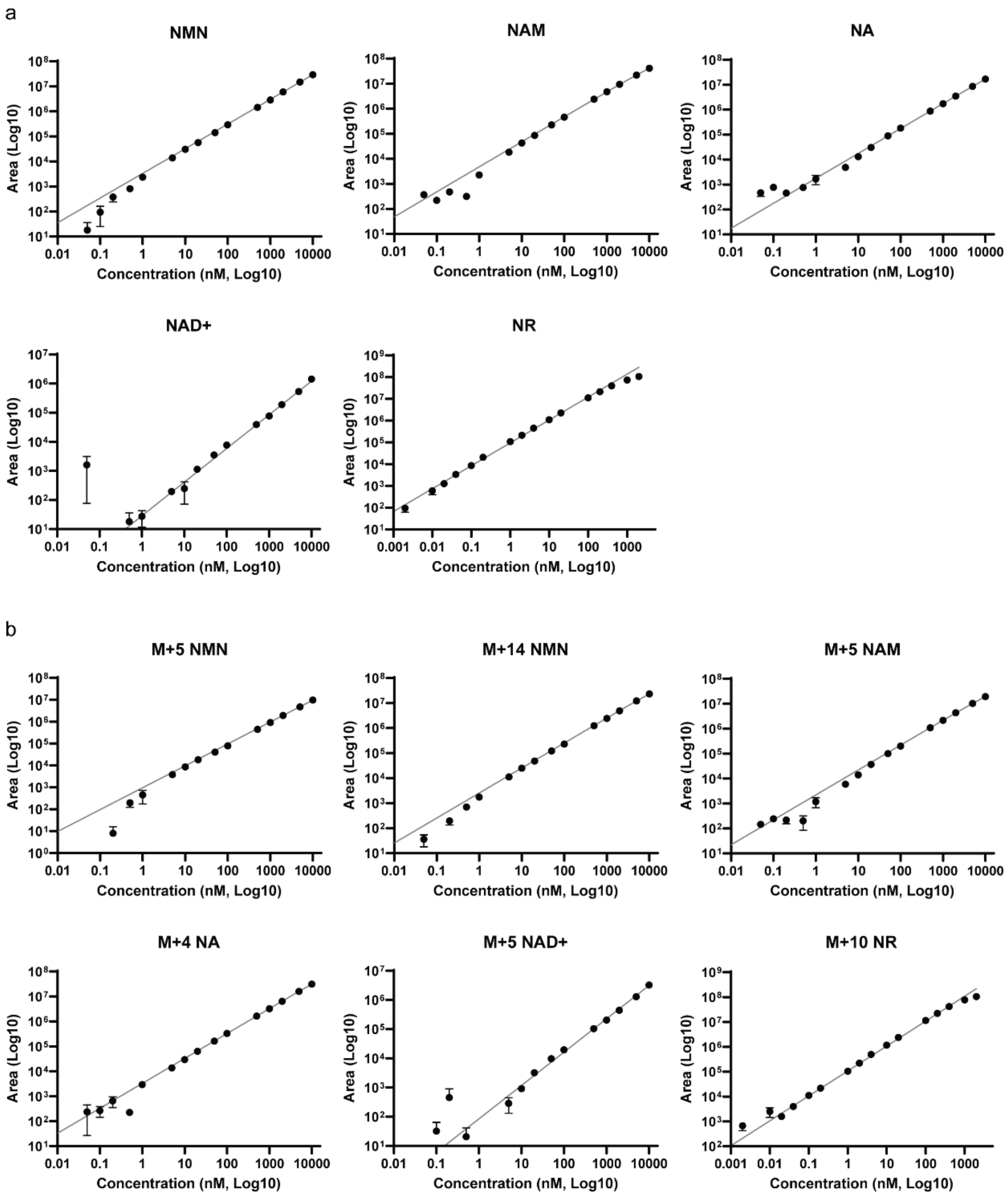

Supplementary Figure 1. Ranges of linearity in each compound.

The area under curves (AUCs) of each regular compound at different ranges of concentrations (0, 0.05, 0.1, 0.2, 0.5, 1, 5, 10, 20, 50, 100, 500, 1000, 2000, 5000, and 10000 nM for NMN, NAD^+, NAM, NA, M+5 NMN, M+5 NAD^+, M+5 NAM, and M+4 NA; 0, 0.002, 0.01, 0.02, 0.04, 0.1, 0.2, 1, 2, 4, 10, 10, 20, 100, 200, 400, 1000, and 2000 nM for NR and M+10 NR) were measured by LC-MS/MS. Areas were plotted with a Log-log line.

Supplementary Figure 2:

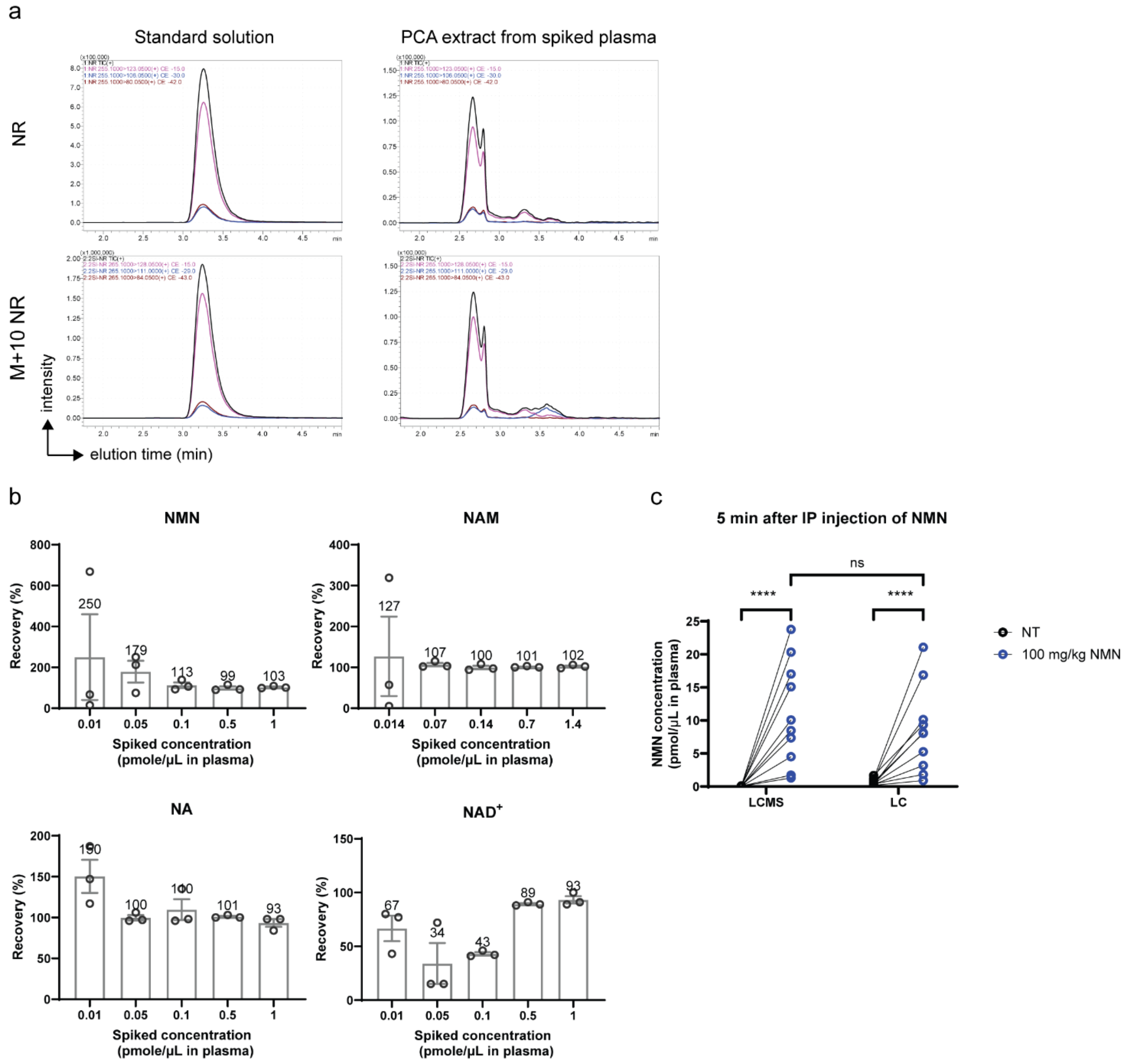

Supplementary Figure 2. Supporting data for LC-MS/MS analysis of PCA extracts and dimeLC-MS/MS analysis of plasma NMN after IP injection.

(a) The peak shape of NR was not similar to standard control after PCA extraction. TICs and transitions were obtained from the MRM of NR and M+10 NR (200 nM standard and PCA extract from spiked plasma) and plotted in a chromatogram. (b) Quantification limit of dimeLC-MS/MS. Regular compounds were added to mouse plasma at the indicated concentration, then concentrations were analyzed by dimeLC-MS/MS. Recovery efficiencies were calculated. Means were calculated from triplicate samples in a single experiment. Data are presented as mean ± SEM. (c) NMN increase after IP injection of 100 mg/kg NMN in each individual mouse. The same data as main figure 2d were used but dots were connected before and after IP injection in the same mice.

Supplementary Figure 3:

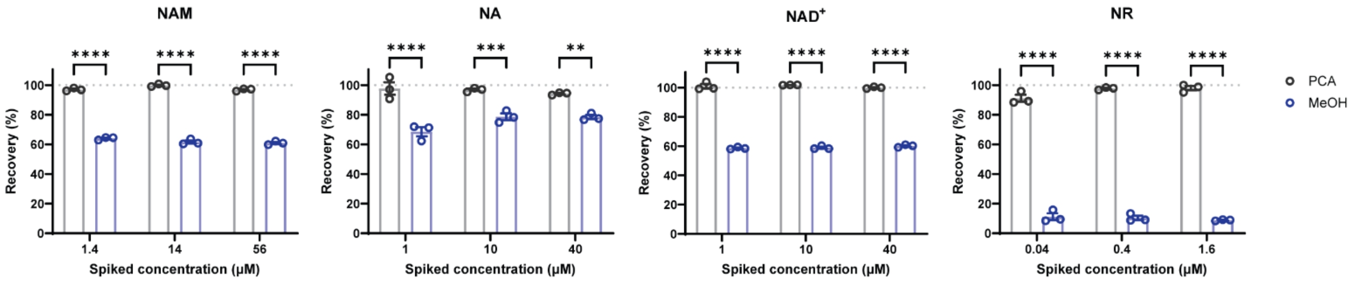

Supplementary Figure 3. Recovery efficiencies of spiked NAM, NA, NAD^+, and NR after PCA and MeOH extraction.

Indicated concentrations of these compounds were added to mouse plasma. After extracting plasma samples with PCA or MeOH-chloroform, each extract was added with corresponding stable isotopic compounds and analyzed with LC-MS/MS. Recovery efficiencies were calculated by an iSTD method. Means were obtained from triplicate samples in a single experiment. Repeated-measures two-way ANOVA was conducted to compare the results with Bonferroni's multiple comparisons *post hoc* test. Data are presented as mean ± SEM. **, p=0.0018; ***, p=0.0006; ****, p<0.0001.

Supplementary Figure 4:

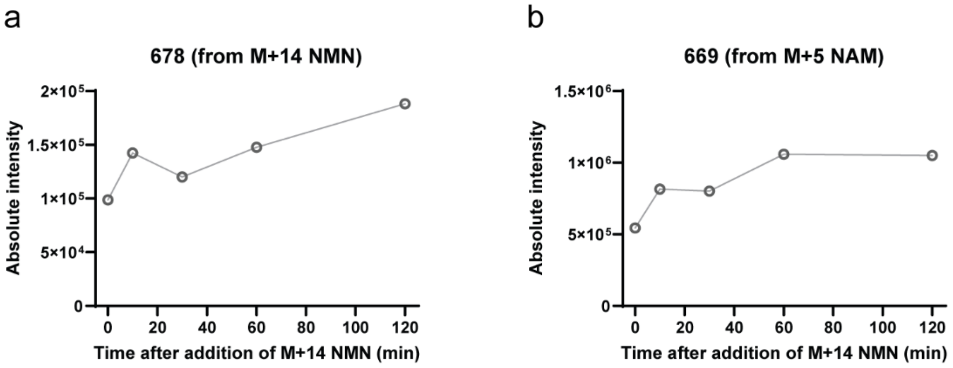

Supplementary Figure 4. NAD+ synthesis from M+14 NMN and M+5 NAM in AML12 cells.

AML12 cells were treated with 200 μM NMN (M+14), and extracted at 10, 30, 60, and 120 min time points after the addition of NMN (M+14). The extract was measured with a positive scan mode. The retention time of NAD^+ was selected and then indicated m/z of NAD^+ ions were analyzed. Absolute intensity was plotted. Data are presented as mean from duplicate measurements of a single sample.
