# Supplementary material for: Absolute quantification of nicotinamide mononucleotide in biological samples by double isotope-mediated liquid chromatography-tandem mass spectrometry (dimeLC-MS/MS)
Source: NPJ Aging. 2024 Jan 2;10(1):2. doi: 10.1038/s41514-023-00133-1 (PMC10762063; doi:10.1038/s41514-023-00133-1)
Supplement: Supplementary file 3 — Reporting Summary [file 41514_2023_133_MOESM3_ESM.pdf]

nature portfolio

Corresponding author(s): Shin-ichiro Imai

Last updated by author(s): Nov 27, 2023

Reporting Summary

Nature Portfolio wishes to improve the reproducibility of the work that we publish. This form provides structure for consistency and transparency in reporting. For further information on Nature Portfolio policies, see our Editorial Policies and the Editorial Policy Checklist.

Statistics

For all statistical analyses, confirm that the following items are present in the figure legend, table legend, main text, or Methods section.

n/a	Confirmed	
☐	☒	The exact sample size (*n*) for each experimental group/condition, given as a discrete number and unit of measurement
☐	☒	A statement on whether measurements were taken from distinct samples or whether the same sample was measured repeatedly
☐	☒	The statistical test(s) used AND whether they are one- or two-sided<br>*Only common tests should be described solely by name; describe more complex techniques in the Methods section.*
☒	☐	A description of all covariates tested
☐	☒	A description of any assumptions or corrections, such as tests of normality and adjustment for multiple comparisons
☐	☒	A full description of the statistical parameters including central tendency (e.g. means) or other basic estimates (e.g. regression coefficient) AND variation (e.g. standard deviation) or associated estimates of uncertainty (e.g. confidence intervals)
☒	☐	For null hypothesis testing, the test statistic (e.g. *F*, *t*, *r*) with confidence intervals, effect sizes, degrees of freedom and *P* value noted<br>*Give P values as exact values whenever suitable.*
☒	☐	For Bayesian analysis, information on the choice of priors and Markov chain Monte Carlo settings
☐	☒	For hierarchical and complex designs, identification of the appropriate level for tests and full reporting of outcomes
☐	☒	Estimates of effect sizes (e.g. Cohen's *d*, Pearson's *r*), indicating how they were calculated

Our web collection on statistics for biologists contains articles on many of the points above.

Software and code

Policy information about availability of computer code

Data collection	LabSolution LCMS (ver.5.98SP1) from Shimadzu Corporation was used to collect mass spec data.
Data analysis	LabSolution LCMS Browse and Analysis (ver.5.98SP1) from Shimadzu Corporation, Microsoft Excel (office 365), and Graphpad Prism (ver.10.1.0) were used for data analysis.

For manuscripts utilizing custom algorithms or software that are central to the research but not yet described in published literature, software must be made available to editors and reviewers. We strongly encourage code deposition in a community repository (e.g. GitHub). See the Nature Portfolio guidelines for submitting code & software for further information.

Data

Policy information about availability of data

All manuscripts must include a data availability statement. This statement should provide the following information, where applicable:

- Accession codes, unique identifiers, or web links for publicly available datasets
- A description of any restrictions on data availability
- For clinical datasets or third party data, please ensure that the statement adheres to our policy

All data generated or analyzed during this study are included in this manuscript. Any additional materials are available from the corresponding author on reasonable request.

Research involving human participants, their data, or biological material

Policy information about studies with human participants or human data. See also policy information about sex, gender (identity/presentation), and sexual orientation and race, ethnicity and racism.

Reporting on sex and gender	We didn't use human samples for our study.
Reporting on race, ethnicity, or other socially relevant groupings	We didn't use human samples for our study.
Population characteristics	We didn't use human samples for our study.
Recruitment	We didn't use human samples for our study.
Ethics oversight	We didn't use human samples for our study.

Note that full information on the approval of the study protocol must also be provided in the manuscript.

Field-specific reporting

Please select the one below that is the best fit for your research. If you are not sure, read the appropriate sections before making your selection.

☒ Life sciences ☐ Behavioural & social sciences ☐ Ecological, evolutionary & environmental sciences

For a reference copy of the document with all sections, see nature.com/documents/nr-reporting-summary-flat.pdf

Life sciences study design

All studies must disclose on these points even when the disclosure is negative.

Sample size	More than 3 independent measurements or 2 independent experiments with three replicates in each experiment were done.
Data exclusions	No data were excluded from the analyses.
Replication	More than 3 independent measurements or 2 independent experiments with three replicates in each experiment were done.
Randomization	We did not use randomization in our study.
Blinding	Blinding was not necessary for our experiments.

Reporting for specific materials, systems and methods

We require information from authors about some types of materials, experimental systems and methods used in many studies. Here, indicate whether each material, system or method listed is relevant to your study. If you are not sure if a list item applies to your research, read the appropriate section before selecting a response.

Materials & experimental systems

n/a	Involved in the study	
☒	☐	Antibodies
☐	☒	Eukaryotic cell lines
☒	☐	Palaeontology and archaeology
☐	☒	Animals and other organisms
☒	☐	Clinical data
☒	☐	Dual use research of concern
☒	☐	Plants

Methods

n/a	Involved in the study	
☒	☐	ChIP-seq
☒	☐	Flow cytometry
☒	☐	MRI-based neuroimaging

Eukaryotic cell lines

Policy information about cell lines and Sex and Gender in Research

Cell line source(s)	AML12 (alpha mouse liver 12) is a mouse hepatocyte cell line originally isolated from the normal liver of a 3 month-old mouse.

Authentication	Based on the description in the ATCC, there are 1,183 citations for this particular cell line.
Mycoplasma contamination	AML12 was purchased from the ATCC (CRL-2254). They were mycoplasma negative.
Commonly misidentified lines (See ICLAC register)	*Name any commonly misidentified cell lines used in the study and provide a rationale for their use.*

Animals and other research organisms

Policy information about studies involving animals; ARRIVE guidelines recommended for reporting animal research, and Sex and Gender in Research

Laboratory animals	C57BL/6J male mice were used. 3-4 month-old or 5-6 month-old mice (young mice) mice and 24-25 month-old mice (old mice) were used.
Wild animals	*Provide details on animals observed in or captured in the field; report species and age where possible. Describe how animals were caught and transported and what happened to captive animals after the study (if killed, explain why and describe method; if released, say where and when) OR state that the study did not involve wild animals.*
Reporting on sex	In this study, we used only male mice to avoid any effect of the estrus cycle in females.
Field-collected samples	*For laboratory work with field-collected samples, describe all relevant parameters such as housing, maintenance, temperature, photoperiod and end-of-experiment protocol OR state that the study did not involve samples collected from the field.*
Ethics oversight	All animal studies were approved by the Institutional Animal Care and Use Committee of Washington University in St. Louis, Missouri, USA (Protocol No. 22-0007), and were in accordance with the National Institutes of Health guidelines.

Note that full information on the approval of the study protocol must also be provided in the manuscript.

Plants

Seed stocks	We didn't use plants for our study.
Novel plant genotypes	We didn't use plants for our study.
Authentication	We didn't use plants for our study.
